# Applicability of Yeast Fermentation to Reduce Fructans and Other FODMAPs

**DOI:** 10.3390/nu10091247

**Published:** 2018-09-06

**Authors:** Vera Fraberger, Lisa-Maria Call, Konrad J. Domig, Stefano D’Amico

**Affiliations:** Department of Food Science and Technology, University of Natural Resources and Life Sciences (BOKU), 1190 Vienna, Austria; vera.fraberger@boku.ac.at (V.F.); lisa.call@boku.ac.at (L.-M.C.); konrad.domig@boku.ac.at (K.J.D.)

**Keywords:** FODMAPs, fructan, IBS, wheat, sourdough, yeasts, fermentation

## Abstract

A diet low in fermentable oligosaccharides, disaccharides, monosaccharides and, polyols (FODMAPs) is recommended for people affected by irritable bowel syndrome (IBS) and non-coeliac wheat sensitivity (NCWS) in order to reduce symptoms. Therefore, the aim of this study was to evaluate the impact of 13 sourdough-related yeasts on FODMAP degradation, especially fructans. First, a model system containing a typical wheat carbohydrate profile was applied to evaluate the growth rate of each yeast strain. Additionally, changes in the sugar composition, for up to four days, were monitored by high-pressure anion-exchange chromatography (HPAEC). A more realistic approach with a wheat flour suspension was used to characterize CO_2_ production according to the Einhorn method. The reduction of the total fructans was analyzed using an enzymatic method. Furthermore, a fingerprint of the present fructans with different degrees of polymerization was analyzed by HPAEC. The results revealed strong differences in the examined yeast strains’ ability to degrade fructans, in both the model system and wheat flour. Overall, *Saccharomyces* *cerevisiae* isolated from Austrian traditional sourdough showed the highest degree of degradation of the total fructan content and the highest gas building capacity, followed by *Torulaspora* *delbrueckii*. Hence, this study provides novel knowledge about the FODMAP conversion of yeast strains.

## 1. Introduction

Current data indicates that irritable bowel syndrome (IBS) affects 7–15% of the population worldwide, with an estimated prevalence of 12% within Europe [1,2]. IBS is the most common chronic gastrointestinal disorder, which is defined as “a functional bowel disorder in which recurrent abdominal pain is associated with defecation or a change in bowel habits” [3]. Symptoms are abdominal pain, flatulence, diarrhea, constipation, intestinal cramps, nausea, and an altered gut microbiota [1,2,3,4,5], leading to a substantial reduction in the health-related quality of life (HRQOL) of patients [2]. Similar symptoms have been reported for people with non-coeliac wheat sensitivity (NCWS). Beside amylase-trypsin inhibitors (ATIs), wheat fructans are suspected to be the main triggers of symptoms [6,7]. Since no biomarkers are thus far available, and indications are similar to other disorders, reliable diagnoses are difficult [8].

However, it has been widely acknowledged that FODMAPs plays a major role in the pathological process of IBS and NCWS [9,10,11]. The term FODMAPs comprises fermentable oligosaccharides (fructans and galactans), disaccharides (lactose), monosaccharides (fructose), and polyols (sorbitol and mannitol), which are present in a number of grains, fruits, vegetables, and dairy products. A lack of enzymes results in the incomplete hydrolysis of glycosidic linkages in these complex polysaccharides, leading to malabsorption. In addition, the poorly absorbed short-chain carbohydrates are osmotically active, drawing more water into the intestine and affecting gut mobility. The undigested polysaccharides are transferred further to the large intestine, where the colonic microbiota rapidly ferments FODMAPs, causing diarrhea and gas production, symptoms consistent with IBS. Therefore, a diet low in FODMAPs has proven to be an effective approach to reduced symptoms in patients with both disorders [8,9,12].

Wheat and its products are staple foods worldwide, accounting for a large extent of the daily consumption of FODMAPs. Moreover, they are the most important source of fructans in the Western European diet. Within FODMAPs, fructans belong to the class of fermentable oligosaccharides, contributing up to 70% to the daily fructan intake [13,14]. Several authors have already stated the influence of FODMAPs, and especially fructans, to trigger the symptoms of IBS and the importance of reducing its concentration in the diet of patients [15,16].

In wheat flour, fructan concentrations range from 1.4–1.7 g/100 g, with whole-wheat flour containing 0.7–2.9 g/100 g [17]. Fifty-percent of fructans exhibit a degree of polymerization (DP) of 3, 4, and 5 (average of 4), with a maximum DP of 14–19, consisting of exclusively or mainly fructose units [14,18], and a maximum of one glucose unit per molecule can be present. Fructans deriving from cereals belong to the graminan type and are branched, which results in more complex structures compared to the inulin-type fructans found in vegetables [17,19].

Due to the high concentrations of fructans in wheat products, patients with IBS and NCWS should consume less FODMAP-containing products, as a single ingestion of approximately 0.3 g/kg can trigger symptoms [20]. However, the authors of Ziegler et al. [21] reported a 77–90% reduction of FODMAPs after yeast proofing for four hours, depending on the flour used. They reported a total reduction of raffinose, whereas fructans were degraded incompletely. Furthermore, this study revealed an excess of fructose. Additionally, Knez et al. [22] demonstrated a 40–60% decrease in fructan content during the bread making process, where the fermentation time, present yeast strain, and yeast counts played a crucial role in the degradation process. The use of sourdough was proven to be one effective method to reduce the content of fructans in bread, as the invertase activity of yeasts degrades fructans [23]. An examination of the impact of traditional sourdough application on the reduction of fructan concentrations in bread showed a decrease of up to 0.06 g/100 g [24].

Lactic acid bacteria and yeasts are present in traditional sourdough at a ratio of 10:1 to 1000:1, whereat these microorganisms lead to superior characteristics of sourdough [25,26,27,28,29]. The most prevalent yeast species in traditional sourdough are *Saccharomyces cerevisiae*, *Candida humilis*, *Wickerhamomyces anomalus*, *Torulaspora delbrueckii*, *Kazachstania exigua*, *Pichia kudriavzevii*, and *Candida glabrata*. Less frequent species include *Kluyveromyces marxianus* and *Hanseniaspora uvarum* [28,30]. Recent investigations [1,31] have already determined the probability of *Kl. marxianus* strain CBS6014 to degrade fructans, showing a 90% decrease of the initial fructan concentration due to dough fermentation for two hours. However, information about the applicability of further sourdough-relevant yeasts to degrade FODMAPs, especially fructans, and a comparison of different yeast strain processes is still lacking.

Therefore, the aim of this study was to investigate the potential of several yeast strains to reduce FODMAP levels, especially fructans. First, a model system with the typical carbohydrates found in wheat was applied to study the degradation of fructans and to monitor the metabolism of other carbohydrates. In addition, a more applied approach with wheat flour was used to obtain data on the gas production and reduction of graminan-type fructans.

## 2. Materials and Methods

### 2.1. Applied Yeast Strains

In total, 13 yeast strains were screened for their specific ability to ferment carbohydrates, especially for their potential to degrade fructans. Three strains were isolated from traditional Austrian sourdoughs and one from a baker’s yeast; eight strains were obtained from either the BCCM or DSMZ databases. Table 1 lists all of the examined strains and their origin. Previously, the isolates obtained from traditional Austrian wheat or rye sourdoughs were identified by partial 26S rDNA sequencing according to Waite et al. [32] and/or by MALDI-TOF MS (matrix-assisted laser desorption/ionization-time of flight mass spectrometry; MALDI Biotyper, Bruker Corporation). For identification by MALDI-TOF MS, the procedure was followed according to the manufacturer’s manual for the extraction method. Table S1 reports the sequencing and MALDI-TOF MS results.

### 2.2. Preparation and Growth of Yeast Cells in a Model System

For the model system, a typical carbohydrate profile found in wheat grains and flours was used. The concentrations of sugars and fructans were set according to the results obtained by Call et al. [33] and are presented in Table 2. To obtain a more realistic profile, sucrose and maltose were added, which are usually included intentionally or are produced by amylases in wheat dough. The appropriate amount of tryptone was dissolved in H_2_O_dest_ and autoclaved at 121 °C for 15 min at one1 bar overpressure. The dissolved carbohydrate solution was filter sterilized through a 0.2-µm polyamide membrane (VWR International GmbH, Darmstadt, Germany) and added to the tryptone solution.

The yeast species were routinely cultivated in worth broth (Merck KGaA, Darmstadt, Germany) and incubated for 48 h at 25 °C. For starvation of the cells, the biomass was transferred to tryptone (6 g/L; Oxoid Ltd., Hampshire, UK) at a dilution ratio of 1:100. After 24 h, the medium, together with starved yeast cultures (1:100) was added in duplicate to Honeycomb format plates (Bartelt GmbH, Graz, Austria). Turbidity data for growth curves of yeast species were analyzed using the automated density monitoring system BioscreenC analyze reader (Oy Growth Curves Ab Ltd., Helsinki, Finland). Reading for 96 h at 25 °C, measurements were taken at an optical density of 600 nm (OD_600_) every 10 min after shaking.

### 2.3. Gas Production Measurement According to Einhorn

The CO_2_ building capacity of yeasts were measured according to the method of Einhorn. Whole-wheat flour from the Austrian variety Arnold was used. Kernels of this variety were collected from nine different locations in Austria (research fields of AGES) over two years (2016 and 2017) to produce a standardized flour. A total of 1 g of flour was mixed with 10 mL of sterilized water and inoculated with the listed yeast strains (Table 1). Yeast counts were in the range of log 7 CFU/mL (detailed yeast counts are presented in Table S2). The yeast-containing wheat flour suspensions were transferred to fermentation locks according to Einhorn. The CO_2_ building capacity was read in mL every 15 min for up to 8 h and 15 min. Figure S1 gives examples of the fermentation locks.

### 2.4. FODMAP Extraction of the Model System

To determine FODMAP conversion in the carbohydrate model system, 4 measuring intervals were defined based on the results of the previous growth experiments—6 h, 24 h, 48 h, and 72 h of incubation. The respective cell mass was transferred from the Honeycomb format plates into 2 mL centrifugation tubes. Proteins were removed by Carrez-precipitation. Hence, 15 µL of Carrez I solution—potassium hexacyanoferrate(II)trihydrate (Merck, Darmstadt, Germany) in 1000 mL H_2_O, and 15 µL of Carrez II Solution—300 g zinc acetate dehydrate (VWR International GmbH, Darmstadt, Germany) in 1000 mL H_2_O was added to precipitate the proteins. After centrifugation at 16,000× *g* for 30 min at 4 °C, the supernatant was transferred to a volumetric flask and filled up to 1 mL. Next, the solution was filtered through a 0.2-µm filter (Rotilabo Mini-Tip syringe filter; Carl Roth GmbH + Co. KG, Karlsruhe, Germany) and diluted to receive carbohydrate concentrations between 5–200 ppm and transferred into vials (1.5 mL clear glass; VWR International GmbH, Darmstadt, Germany) for further analysis.

### 2.5. Extraction of Graminan-Type Fructans

To determine changes of DP of graminan-type fructans after fermentation, a fingerprint analysis was performed after extraction of the wheat flour-water suspension. Following fermentation for 8 h and 15 min, the suspensions in the Einhorn locks were subjected to an ethanol extraction. The matrix was transferred into a centrifugation tube, 30 mL of 96% ethanol (VWR International GmbH, Darmstadt, Germany) was added and the mixture was heated to 80 °C for 20 min. After centrifugation at 9000× *g* for 6 min, the supernatant was filled up to the defined volume of 50 mL. A 3 mL aliquot of the extract was evaporated at 50 °C under a nitrogen stream to dryness and resuspended in 0.96 mL of water. Twenty µL each of amyloglucosidase (AMG) and alpha-amylase (Novozymes, Ireland) were added, and incubation was carried out at 50 °C for 30 min. Afterwards, protein precipitation, as described in Section 2.4, was conducted. The solution was sterile-filtered through a 0.2-µm filter (Rotilabo Mini-Tip syringe filter; Carl Roth GmbH + Co. KG, Karlsruhe, Germany) and used further for HPAEC-PAD (high pressure anion-exchange chromatography-pulsed amperometric detection) analysis.

### 2.6. FODMAP and Fructan Analysis

To determine the FODMAP concentrations and the DP of fructans, HPAEC with a Carbopac PA210 column (2 × 150 mm) from ThermoFisher Scientific (Sunnyvale, CA, USA) was applied at a flow rate of 0.15 mL/min. A gradient elution with 150 mM NaOH and 150 mM NaOH/500 mM Na-acetate was used on a DionexTM ICS-5000+ System (ThermoFisher Scientific, Sunnyvale, CA, USA) for separation. The concentrations of glucose, fructose, sucrose, maltose, raffinose, and fructans were determined by pulsed amphoteric detection (PAD). A gold electrode with a carbohydrate waveform was used for electrochemical detection according to the manufacturer’s instructions. For integration and calibration, the software Chromeleon 7 was utilized. The method was calibrated in a range from 0.1–25 mg/L with glucose, fructose, sucrose, maltose, raffinose (≥99.5% purity; Carl Roth GmbH + Co. KG, Karlsruhe, Germany), and small fructans from DP 3–5 (Megazyme, Bray, UK). For comparison, a fructan standard with up to DP 8 was also analyzed (fructooligosaccharide P-FOS28, Megazyme, Bray, UK). The qualitative reduction of the total graminan-type fructans, present in the wheat flour suspensions, was determined by an enzymatic-spectrophotometric method according to AOAC standard 999.3.

### 2.7. Statistical Analysis

The data interpretation was performed with SPSS software (IBM). The correlation test according to Pearson was applied to compare the fructan conversion results gained by the enzymatic assay and HPAEC-PAD.

## 3. Results

### 3.1. Yeast Growth

**Figure 1:**
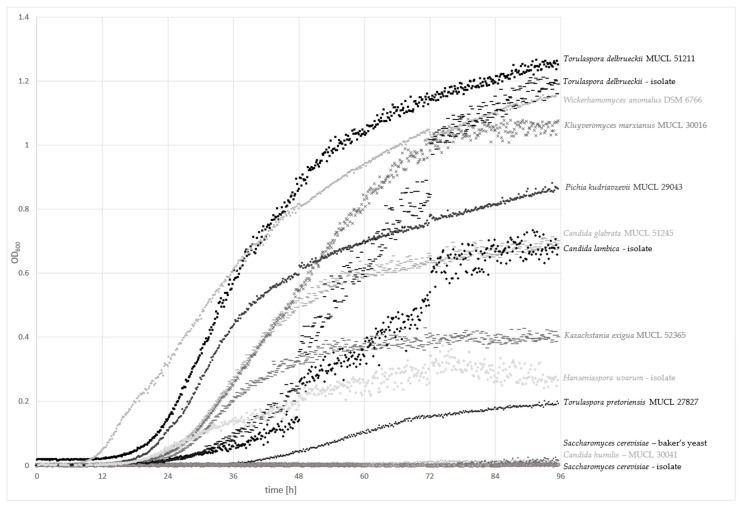
Measurement of the optical density at 600 nm (OD_600_) over 96 h of incubation at 25 °C to indicate the growth rate of sourdough-relevant yeasts in a model medium containing mono-/di-/trisaccharides, and fructans, according to the average concentrations found in 20 Austrian wheat varieties (Table 1).

The cell growth of 13 different yeast strains were evaluated for up to 96 h at 25 °C with an automated density monitoring system using a model medium, containing a typical carbohydrate profile found in whole-grain wheat. The growth curves are plotted in Figure 1.

*Torulaspora delbrueckii* strains (MUCL 51211 and the sourdough isolate) exhibited the highest optical density at 600 nm (OD_600_) of 1.3–1.2 nm, respectively, after 96 h of incubation, followed by *Wickerhamomyces anomalus* DSM 6766 and *Kluyveromyces marxianus* MUCL 30016. *Pichia kudriavzevii* MUCL 29043, *Candida glabrata* MUCL 51245, and the sourdough isolate *C. lambica* showed OD_600_ values ranging from 0.9–0.7 nm. *Kazachstania exigua* MUCL 52365, the isolate *Hanseniaspora uvarum*, and *Torulaspora pretoriensis* MUCL 27827 exhibited values between 0.4 and 0.2 nm. Only *Saccharomyces cerevisiae* isolates (baker’s yeast and the sourdough isolate) and *C. humilis* MUCL 30041 showed no significant increase in the OD_600_.

A lag phase shorter than 12 h was only achieved by *W. anomalus* DSM 6766. Exponential growth after 18–20 h was observed for *T. delbrueckii* and *P. kudriavzevii* MUCL 29043. The other strains started the log phase after a period of more than 20 h. *T. pretoriensis* MUCL 27827 showed slow growth, and a moderate increase of OD_600_ was detected after over 36 h. Generally, the strains with the fastest growth rates had the highest OD_600_ values. Nevertheless, some exceptions were observed. *T. delbruecki*i isolated from sourdough achieved high final OD_600_ values, although the log phase started after approximately 24 h. *K. exigua* MUCL 52356 accomplished the lag phase after about 20 h, whereas only medium-range OD_600_ values were achieved after 96 h. *H. uvarum* exhibited similar growth behavior, but the final OD_600_ was low in comparison to *K. exigua* MUCL 52356.

### 3.2. CO_2_ Formation Properties

**Figure 2:**
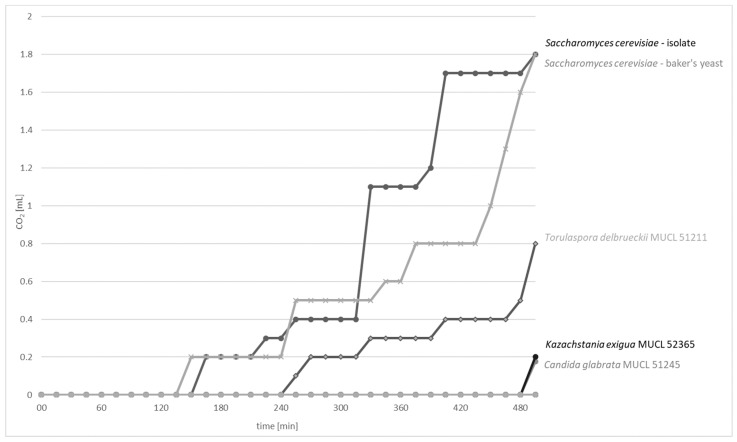
Time course of CO_2_ production (mL CO_2_/min) of CO_2_-positive yeast strains *S. cerevisiae*, *T. delbrueckii* MUCL 51211, *K. exigua* MUCL 52365, and *C. glabrata* MUCL 51245 over 8 h and 15 min of incubation at 25 °C.

The CO_2_ production capacity of 13 yeast strains were tested using fermentation locks according to Einhorn (Figure S1). The data on the CO_2_ production rates (mL CO_2_/min) of CO_2_-positive yeast strains are presented in Figure 2. *S. cerevisiae* strains exhibited the highest CO_2_ formation capacity (1.8 mL) after 8 h and 15 min of incubation at 25 °C, followed by *T. delbrueckii* MUCL 51211 with a CO_2_ production of 0.8 mL. *K. exigua* MUCL 52365 and *C. glabrata* MUCL 51245 exhibited poor formation capacities of 0.2 mL. Further tested strains *C. humilis* MUCL 30041, *Kl. marxianus* MUCL 30016, *P. kudriavzevii* MUCL 29043, *T. pretoriensis* MUCL 27827, *W. anomalus* DSM 6766, and sourdough isolates *P. fermentans*, *C. lambica*, *T. delbrueckii,* and *H. uvarum* had no observable CO_2_ production.

For CO_2_-positive yeast strains, the graphs showed mainly constant levels during the period of 30–90 min, followed by an observable strong and sudden increase. This effect can be explained by the production of small CO_2_ bubbles, which were first entrapped within the wheat flour suspension. After they have aggregated to bigger bubbles, the volume of CO_2_ was measureable as the bubbles were able to displace the suspension and rise to the top. However, although the curves of the *S. cerevisiae* strains looked different, similar gas productions can be assumed because of these circumstances.

### 3.3. Conversion of Carbohydrates by Yeasts in the Model System

**Figure 3:**
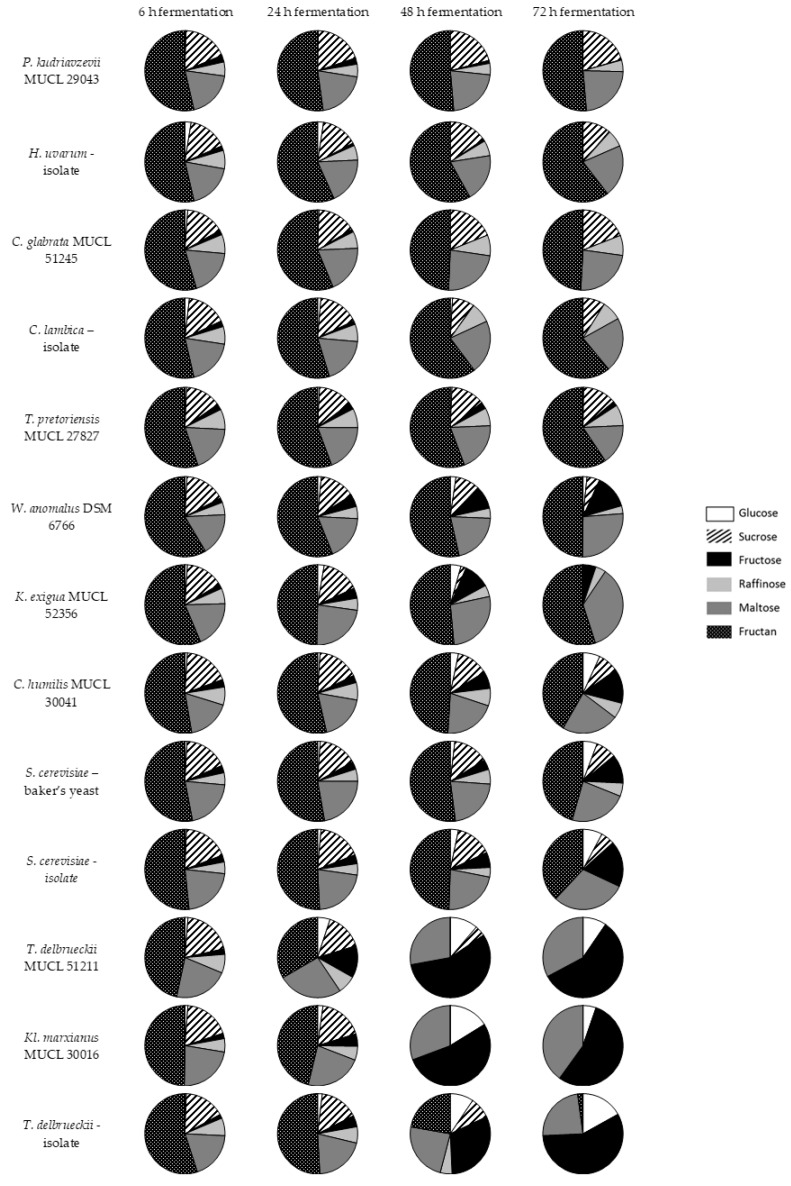
Fermentation profile of 13 yeasts strains over 4 measuring points, 6 h, 24 h, 48 h, and 72 h of incubation at 25 °C in a model system; examined carbohydrates: Glucose, sucrose, fructose, raffinose, maltose, and fructan.

The fermentation profile of 13 different yeast strains were examined over three days. After four different time periods (6 h, 24 h, 48 h, and 72 h), samples were taken and analyzed to evaluate changes in the carbohydrate profile, i.e., the degradation or production of glucose, sucrose, fructose, raffinose, maltose, and fructans (DP 4–8). The results are illustrated in Figure 3 as pie charts, showing the relative abundance of quantified sugars and fructans based on HPAEC-PAD analysis.

Of the 13 tested yeasts, *P. kudriavzevii* MUCL 29043, *C. glabrata* MUCL 51245, *T. pretoriensis* MUCL 27827, and the sourdough isolates *H. uvarum* and *C. lambica* were not able to reduce fructan levels to a relevant degree. Furthermore, the concentration of raffinose remained constant, which indicted that these strains were not able to consume this trisaccharide. The small amount of initial glucose were digested quite fast, and fructose slightly slower. Among these strains, only *T. pretoriensis* MUCL 27827 showed fructose residues after three days. The maltose concentrations remained almost unchanged. Sucrose was fermented to a small degree by *T. pretoriensis* MUCL 27827, *H. uvarum* and *C. lambica*, whereas sucrose levels were not significantly affected by the other yeast strains of this group.

A second group consisted of strains with moderate fructan conversion including *K. exigua* MUCL 52356, *C. humilis* MUCL 30041, W*. anomalus* DSM 6766, and both *S. cerevisiae* strains. These yeasts were able to degrade fructans. However, after three days of fermentation, high fructan levels still remained. Raffinose was degraded only partially by the examined yeast strains, with one exception—the isolate of *S. cerevisae* completely converted this trisaccharide. Due to depolymerisation of the oligosaccharides, the content of monosaccharides had risen. In particular, the concentration of fructose increased markedly, and glucose increased to a lesser extent. Maltose was not fermented in notable amounts and remained more or less constant. Due to the reduction of other sugars, the relative abundance of maltose could actually increase, as was illustrated for *K. exigua* MUCL 52356. Sucrose was fermented incompletely, as low sucrose concentrations were detected after three days.

Both of the *T. delbrueckii* strains, and *Kl. marxianus* MUCL 30016 demonstrated the best fructan degradation. In addition, the highest amounts of fructose were produced by these strains. *Kl. marxianus* MUCL 30016 and *T. delbrueckii* MUCL 51211 were able to completely ferment fructans after two days; a reduction in the fructose and glucose content from 48–72 h of incubation was observable for these yeasts as well. The *T. delbrueckii* isolate degraded fructans more slowly, and small fructans residues were detected. Raffinose and sucrose were digested entirely. Maltose was fermented only to a minor extent and persisted more or less unchanged. Again, due to the reduction of other sugars, a relative increase in maltose was seen, which can be explained by presenting the relative abundance as pie charts.

### 3.4. Fructan Degradation by Yeasts in Wheat Flour

**Figure 4:**
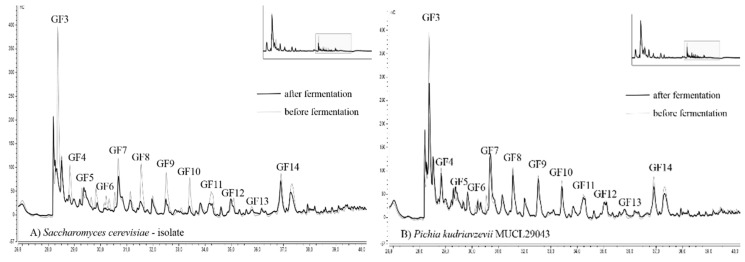
HPAEC (high pressure anion-exchange chromatography) fingerprint of fructans fermented by (**A**) *Saccharomyces cerevisiae*–sourdough isolate, and (**B**) *Pichia kudriavzevii* MUCL 29043. Chromatograms showing the initial fructan profile (grey line) and the alteration after 8 h and 15 min of fermentation at 25 °C (black line) and are illustrated for both strains. G (glucose) and F (fructose).

Chromatograms of fructan fingerprints, showing the distribution of DP, from two selected strains with the best and worst ability for fructan degradation are presented in Figure 4. The overall results of the 13 yeast strains analyzed by HPAEC-PAD are available within the supplementary material (Figure S3).

To evaluate the alteration of the fructan profile ranging from DP 4–16, the fingerprint before and after fermentation is illustrated. The DP of fructans was estimated based on their retention times according to Verspreet et al. [34]. Due to the removal of starch-based dextrins by amylases, all detected peaks can be assigned to fructans.

*C. humilis* MUCL 30041, *C. glabrata* MUCL 51245, and *P. kudriavzevii* MUCL 29043 exhibited the lowest degree of alteration compared to the initial fructan fingerprint. The adulteration of fructans, as shown by HPAE chromatography, was very low. Only the smallest fructans with DP 4 were reduced in noticeable amounts. The height of all other peaks was reduced only to a small degree. *T. delbrueckii* MUCL 51211, *S. cerevisiae*–baker’s yeast, *Kl. marxianus* MUCL 30016, *W. anomalus* DSM 6766, *T. pretoriensis* MUCL 27827, and the sourdough isolates *C. lambica* and *H. uvarum* exhibited moderate degradation of fructans. The most significant changes were observed for *K. exigua* MUCL 52365 and the sourdough isolates *S. cerevisiae* and *T. delbrueckii* (Figure 4). Fingerprints of these strains revealed severe reductions of peaks until DP 11 (GF 10). The fructans with higher molecular weights showed less adulteration in peak height. These findings were confirmed by other studies, which reported that smaller fructans were consumed more efficiently than those with higher DP [35].

**Figure 5:**
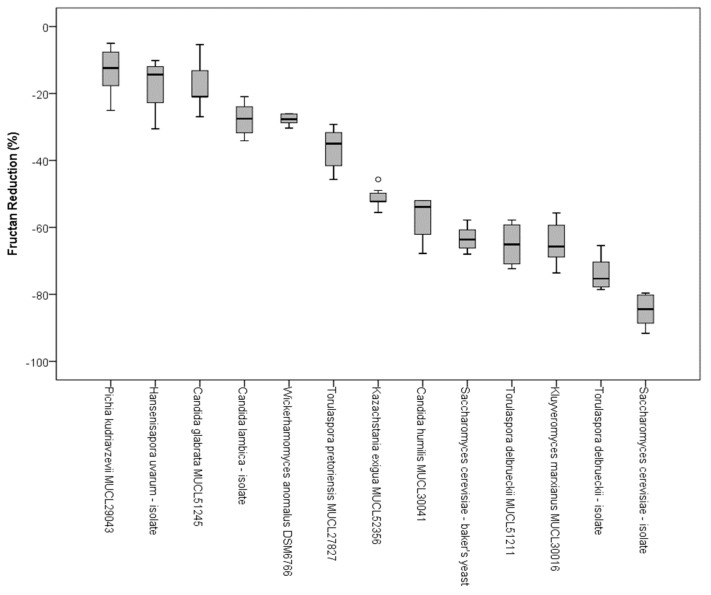
Box plots of the fructan reduction of examined yeast strains in wheat flour after 8 h and 15 min of incubation at 25 °C, determined according to the AOAC 999.3 standard; circles present outliners.

In addition to the qualitative characterization of fructans by HPAEC, the samples from the CO_2_ production tests were analyzed by an enzymatic method to evaluate the decrease of fructan concentration. The reduction of total fructan content after 8 h and 15 min of incubation at 25 °C was measured according to AOAC standard 999.3. To calculate the decrease of fructose oligomers, the initial content and the amount of fructans after fermentation were quantified. The reductions in fructan content (%) due to fermentation of single yeast strains are presented in Figure 5. With a decrease of the total fructan content of 10–30%, *Candida* spp., *H. uvarum*, *T. pretoriensis* MUCL 27827, and *W. anomalus* DSM 6766 showed the lowest capacity for FODMAP reduction of the tested yeast species. *K. exigua* MUCL 52365 revealed a total fructan reduction of approximately 50%. *S. cerevisiae*-baker’s yeast, the *T. delbrueckii* stains, and *Kl. marxianus* MULC 30016 revealed a decrease in the total fructan content between 60–80%. With a reduction of 83%, the sourdough isolate of *S. cerevisiae* had the highest decrease in fructans.

## 4. Discussion

This study provides novel findings regarding the FODMAP reduction during fermentation by yeasts. First, the degradation potential of 13 yeast strains related to traditional sourdough were evaluated using a model system containing small fructooligosaccharides (max. DP 8) from chicory. Second, the alteration of graminan-type fructans in wheat flour was investigated using a yeast-mediated fermentation procedure. For examination of the leavening capacity, the CO_2_ building potential was determined according to Einhorn.

### 4.1. Conversion Dynamics of Carbohydrates by Yeasts in the Model System and in Wheat Flour

An assessment of the degradation and consumption of saccharides in a model system revealed very different capacities of the examined yeast strains. The chosen sugar profile, together with short-chain fructans (until DP 8), was suitable for the evaluation of fructan degradation and consumption of sugars found in wheat flour. Due to the simple fructan profile, a fast and accurate quantification by HPAEC was possible. The addition of maltose and sucrose provided a realistic system that closely matched atypical wheat dough. The established model system classified the examined yeast strains into three groups: Yeasts with poor, moderate, and superior fructan degradation. Similar results were obtained for the fermentation experiments of CO_2_ production within a wheat flour suspension. Again, three different groups regarding the degree of fructan hydrolysis could be defined.

Correlation analysis according to Pearson revealed a high consistency between the two systems. A highly significant (*p*-level of 0.01) relationship with an *R*^2^ of 0.59 was observed between the enzymatically measured fructan hydrolysis in wheat flour and the remaining fructan levels in the model system after fermentation for 72 h. Obviously, a stronger correlation was prevented due to the different time periods of fermentation (the model system was 72 h compared to approximately 8 h for the fermentation experiments with wheat flour). A second reason for the low correlation could be the different growth rates of the examined yeast strains, especially with respect to both *S. cerevisiae* strains. These strains showed very low OD_600_ values and thus poor growth. Furthermore, fructans were hydrolysed to a lower extent, compared to the setup with wheat flour. Excluding the data from both of the *S. cerevisiae* strains revealed an increased correlation, with an R^2^ of 0.83 between the fermented wheat flour suspension and the model system.

Moreover, the conversion and consumption of other sugars could be evaluated by the model system. Each strain fermented sucrose, whereat for *C. glabrata* MUCL 51245, the lowest sucrose decrease of 26% was observed. One exception was *P. kudriavzevii* MUCL 29043, where an increased concentration of 6% was measured due to partial fructan hydrolysis. These results were in accordance with previous studies [36]. Raffinose was metabolized by the majority of the examined yeasts. Some strains showed no or only minor breakdown of raffinose, which could arise from small variations due to the determination method used. A comparison with the literature verified this assumption for *Candida* spp. and *P. kudriavzevii* [36]. Controversial findings were found in respect to *K. exigua* [36]. Furthermore, relationships between fructan degradation and the consumption of raffinose and sucrose were found. Yeast strains, which depolymerized fructans to a large extent, consumed raffinose and sucrose as well, and invertase and inulinase are responsible for releasing the fructose moiety of these carbohydrates [37].

According to the literature [36], each tested yeast strain is able to ferment glucose, which was confirmed by this study as well. However, the consumption was partly masked due to the production of glucose, as it was released during the decomposition of fructans, sucrose, and raffinose. This effect was also observable for fructose concentrations, which increased as the fructans and raffinose were degraded. However, for species exhibiting the highest potential to depolymerize fructans (*T. delbrueckii* MUCL 51211 and *Kl. marxianus* MUCL 30016), a decrease in fructose was apparent after 72 h of incubation. This is consistent with studies showing the capacity of *T. delbrueckii* and *Kl. marxianus* to degrade fructose [38,39].

As previously mentioned, invertase and inulinase are able to depolymerize fructans. However, large polysaccharides barely pass the cell wall and thus must be hydrolysed outside of the cell [40]. The invertase produced by *S. cerevisiae* is retained inside the cell wall, whereas inulinase, produced by *Kl. marxianus,* is partly secreted [40]. This might explain why *Kl. marxianus* degrades fructans faster and to a higher degree than *S. cerevisiae*. The chosen system and sugar concentrations were not suitable to evaluate maltose conversion, because other sugars were present in excess during the evaluated time period. Nevertheless, maltose-positive strains [36], such as baker’s yeast, *T. delbrueckii*, and *W. anomalus*, showed a decrease in maltose concentration over 72 h of incubation.

Furthermore, the detected range of fructan DP (up to 15) was similar to the results reported in other studies. An average DP of five makes up 50% of the total fructan content in whole-wheat flour, with reported values up to DP 15 [14], whereas Haskå, Nyman, and Andersson [18] found the highest DP of 19. The fingerprint analysis of graminan-type fructans supported the results. Strains with a high degree of fructan hydrolysis showed intensively reduced peak heights in the HPAE chromatograms. Since high molecular weight fructans affect the enzymatic quantification stronger than smaller ones, only a moderate correlation can be expected.

### 4.2. Fructan Degradation Potential of Different Yeast Strains

For *T. delbrueckii* and *Kl. marxianus* MULC 30016, the highest potential to degrade fructans was observed in the model system and in the whole-wheat flour fermentation experiment. These findings are consistent with previous results [1] that have reported a 90% degradation of fructans by *Kl. marxianus* CBS6014 during bread making. In addition, *Kl. marxianus* MULC 30016 exhibits the potential to degrade fructose, which is released due to fructan depolymerisation to fructose and glucose. As described in other studies [31], *S. cerevisiae* showed a decreased potential to ferment fructans compared to the already mentioned yeast species. These findings concur with those of our study, as *S. cerevisiae* isolated from Austrian traditional sourdough had a degradation potential of 18% within 6 h of incubation, whereas after 72 h, a decrease of 54% in the fructan content was achieved within the model system. However, within the whole-wheat flour experimental setup, a decrease in total fructans (until DP 15) of up to 83% was determined. As discussed previously, the marginal growth should be responsible for a low fructan conversion, whereas inoculation with similar yeasts counts inhibited this problematic circumstance.

In contrast, other sourdough-relevant yeasts proved to be less applicable in reducing FODMAPs in the model system and in the whole-wheat flour experiment. In particular, *P. kudriavzevii* MUCL 29043, *C. glabrata* MUCL 51245, *W. anomalus* DSM 6766, *T. pretoriensis* MUCL 27827, and the sourdough isolates *H. uvarum* and *C. lambica* showed poor fructan degradation.

## 5. Conclusions

This study evaluated the FODMAP degradation ability of different typical sourdough yeasts in a simplified model and when using wheat flour as a matrix. Both systems were able to identify differences between the examined strains and showed high consistency. Only the poor growth of *S. cerevisiae* inhibited a higher reliability of the model system. Furthermore, the results regarding the conversion of other FODMAPs such as raffinose were generated by the model system. The fingerprints of fructans measured by HPAEC before and after fermentation supported the fructan degradation results. A comparison with data from other studies confirmed the findings of this study. Wheat bakery products with low FODMAP content might be suitable for IBS patients, but probably only to a very limited degree for individuals suffering from NCWS. The role of ATIs (amylase/trypsin inhibitors) in wheat products must also be considered for people with NCWS [6,7]. In addition, further studies are needed to demonstrate the degradation of FODMAPs in the consumed product, in this case, sourdough fermented bread.

In summary, the results of this study clearly demonstrated the potential of several yeast strains, especially *S. cerevisiae* and *T. delbrueckii*, isolated from traditional sourdough, to strongly reduce FODMAPs, and particularly, the fructan content during fermentation. Furthermore, these yeasts exhibited superior CO_2_ production, which revealed their potential to produce wheat bread with improved leavening characteristics. Therefore, this study might aid to explore the potential of several yeasts to produce a low FODMAP diet with palatable food. However, typical sourdoughs contain a wide range of different lactic acid bacteria and yeasts. Thus, further research on their synergistic effects should be conducted. In particular, the ability of LAB to lower pH, thus supporting yeast invertase activity, is of great interest [17]. Summing up, the purpose of this study was to generate basic data about the potential of single yeast strains to reduce FODMAPs. Based on our findings, more applied research studies should be performed to produce wheat breads suitable for patients with IBS or NCWS.

## Figures and Tables

**Table 1:** Yeast species, strain information, and source of isolation used for the determination of the potential to degrade FODMAPs.

Species	Strain	Source of Isolation
*Candida glabrata*	BCCM/MUCL ^1^ 51245	wheat sourdough, mill D12
*Candida humilis*	BCCM/MUCL ^1^ 30041	San Francisco sourdough
*Kazachstania exigua*	BCCM/MUCL ^1^ 52365	brewing, Japan
*Kluyveromyces marxianus*	BCCM/MUCL ^1^ 30016	-
*Pichia kudriavzevii*	BCCM/MUCL ^1^ 29043	industrial sourdough, France
*Torulaspora delbrueckii*	BCCM/MUCL ^1^ 51211	wheat and rye sourdough, artisan bakery, Belgium
*Torulaspora pretoriensis*	BCCM/MUCL ^1^ 27827	soil, South Africa
*Wickerhamomyces anomalus*	DSM ^2^ 6766	-
*Candida lambica*	isolate	rye sourdough, Austria
*Hanseniaspora uvarum*	isolate	wheat sourdough, Austria
*Saccharomyces cerevisiae*	isolate	baker’s yeast
*Saccharomyces cerevisiae*	isolate	rye sourdough, Austria
*Torulaspora delbrueckii*	isolate	wheat sourdough, Austria

^1^ BCCM/MUCL: Belgian Coordinated Collection of Microorganisms/Agro-food and Environmental Fungal Collection. ^2^ DSMZ: Leibniz-Institute DSMZ-German Collection of Microorganisms and Cell Cultures.

**Table 2:** Medium composition of the model system [33].

	Ingredient	Supplier	[g/100 mL]
Natural ratios occurring in flour	d-Glucose	Carl Roth GmbH + Co. KG, Karlsruhe, Germany	0.02
	d-Fructose		0.04
	Sucrose		0.41
	Raffinose pentahydrate		0.17
	Maltose monohydrate		0.04
	Fructooligosaccharides from chicory (max. DP 8)	Megazyme, Bray, Ireland	1.37
Supplements	Maltose monohydrate	Carl Roth GmbH + Co. KG, Karlsruhe, Germany	0.64
	Sucrose		0.4
	Tryptone	Oxoid LTD, Hampshire, England	0.6

## Supplementary Materials

Supplementary materials are available online at http://www.mdpi.com/2072-6643/10/9/1247/s1. Table S1: Utilized yeast species, source of isolation, and determination of identification by MALDI-TOF MS and % similarity with the accession number of the closest relative by blastn received by 16S rDNA sequencing, Table S2: Determination of log CFU g^−1^ of 13 different yeast strains on YG-agar (Merck, Darmstadt, Germany), with incubation conditions of 25 °C for 48 h, Figure S1: Determination of the gas building capacity of (A) *Saccharomyces cerevisiae*, (B) *Torulaspora delbrueckii* MUCL 51211 and (C) *Kluyveromyces marxianus* MUCL 30016 by fermentation locks according to Einhorn, Figure S2: HPAEC-PAD analysis with CarboPac PAD 210 250 × 2 mm and eluent A (100 mM NaOH) and B (100 mM NaOH/500 mM NaAcetate) to determine glucose, sucrose, fructose, raffinose, maltose, and fructans (max. DP 8) metabolism by *Kluyveromyces marxianus* MUCL 30016 at point 0, after 6 h, 24 h, 48 h, and 72 h of fermentation, Figure S3: HPAEC-PAD analysis of (A) *Candida humilis* MUCL 30041 and (B) *Candida glabrata* MUCL 51245, and (C) *Pichia kudriavzevii* MUCL 29043 showing the initial fructan presence (black line) and after 495 min of fermentation at 25 °C (blue line), with a low conversion rate of fructan, Figure S4: HPAEC-PAD fructan fingerprint analysis of (A) *Torulaspora delbrueckii* MUCL 51211, (B) *Saccharomyces cerevisiae–*baker’s yeast, (C) *Kluyveromyces marxianus* MUCL 30016, (D) *Wickerhamomyces anomalus* DSM 6766, (E) *Torulaspora pretoriensis* MUCL 27827, (F) *Candida lambica*–sourdough isolate, and (G) *Hanseniaspora uvarum*–sourdough isolate, showing the initial fructan presence (blue line) and after 8 h 15 min of fermentation (black line) at 25 °C, with moderate degradation of fructans, Figure S5: HPAEC-PAD analysis of (A) *Kazachstania exigua* MUCL 52365, (B) *Saccharomyces cerevisiae*-sourdough isolate, and (C) *Torulaspora delbrueckii*-sourdough isolate, showing the initial fructan presence (blue line) and after 8 h 15 min of fermentation (black line) at 25 °C, with the highest degradation of fructans.
