# Supplementary material for: Applicability of Yeast Fermentation to Reduce Fructans and Other FODMAPs
Source: Nutrients. 2018 Sep 6;10(9):1247. doi: 10.3390/nu10091247 (PMC6163668; doi:10.3390/nu10091247)
Supplement: Supplementary file 1 [file nutrients-10-01247-s001.pdf]

nutrients

MDPI

Supplementary Materials:

Table S1: Utilized yeast species, source of isolation, and determination of identification by MALDI-TOF MS and % similarity with the accession number of the closest relative by blastn received by 16S rDNA sequencing

Yeast species	Isolation source	MALDI-TOF MS score	% similarity (accession no. of the closest relative by blastn)
Hanseniaspora uvarum	wheat sourdough, Austria	2.30	n.a.[1]
Saccharomyces cerevisiae	baker's yeast	2.03	n.a.[1]
Saccharomyces cerevisiae	rye sourdough, Austria	2.13	99 (KX428529.1)
Torulaspora delbrueckii	wheat sourdough, Austria	1.65*	99 (MH010872.1)

[1] n.a.: not analyzed; *score less than 2.0 indicates ambiguous identification;

Table S2: Determination of log CFU g^{-1} of 13 different yeast strains on YG-agar (Merck, Darmstadt, Germany), with incubation conditions of 25 °C for 48 h.

Species	Strain	log cfu/mL
Candida glabrata	BCCM/MUCL[1] 51245	7.9
Candida humilis	BCCM/MUCL[1] 30041	6.9
Kazachstania exigua	BCCM/MUCL[1] 52365	7.3
Kluyveromyces marxianus	BCCM/MUCL[1] 30016	7.1
Pichia kudriavzevii	BCCM/MUCL[1] 29043	7.2
Torulaspora delbrueckii	BCCM/MUCL[1] 51211	7.8
Torulaspora pretoriensis	BCCM/MUCL[1] 27827	7.6
Wickerhamomyces anomalus	DSM[2] 6766	7.2
Hanseniaspora uvarum	sourdough isolate	7.1
Saccharomyces cerevisiae	sourdough isolate	7.6
Saccharomyces cerevisiae	baker's yeast	7.3
Torulaspora delbrueckii	sourdough isolate	7.7

[1] BCCM/MUCL: Belgian Coordinated Collection of Microorganisms/Agro-food & Environmental Fungal Collection. [2] DSMZ: Leibniz-Institute DSMZ-German Collection of Microorganisms and Cell Cultures.

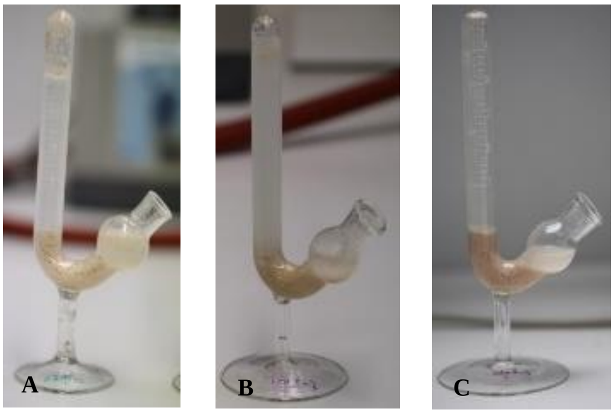

Figure S1: Determination of the gas building capacity of (**A**) *Saccharomyces cerevisiae*, (**B**) *Torulaspora delbrueckii* MUCL 51211 and (**C**) *Kluyveromyces marxianus* MUCL 30016 by fermentation locks according to Einhorn.

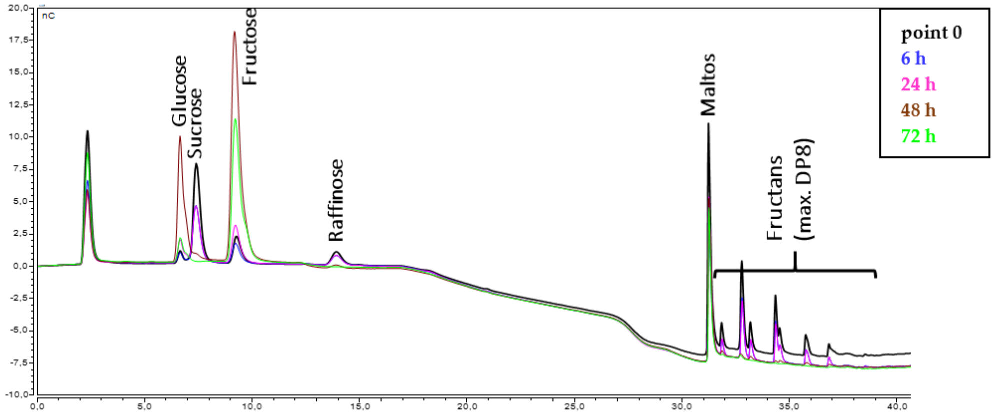

Figure S2: HPAEC-PAD analysis with CarboPac PAD 210 250x2 mm and eluent A (100 mM NaOH) and B (100 mM NaOH/500 mM NaAcetate) to determine glucose, sucrose, fructose, raffinose, maltose, and fructans (max. DP 8) metabolism by *Kluyveromyces marxianus* MUCL 30016 at point 0, after 6 h, 24 h, 48 h, and 72 h of fermentation.

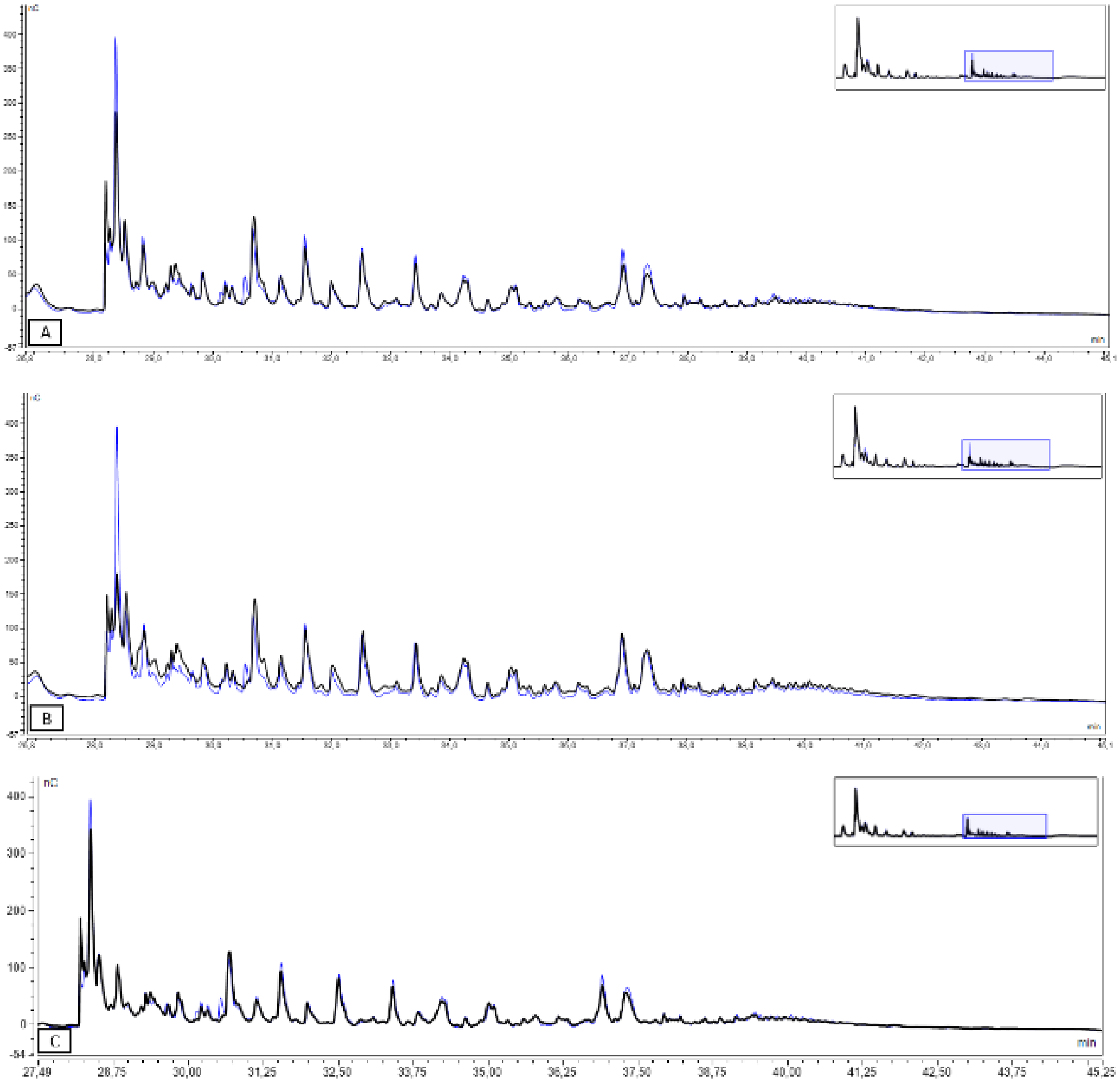

Figure S3: HPAEC-PAD analysis of (**A**) *Candida humilis* MUCL 30041 and (**B**) *Candida glabrata* MUCL 51245, and (**C**) *Pichia kudriavzevii* MUCL 29043 showing the initial fructan presence (black line) and after 8 h 15 min of fermentation at 25 °C (blue line), with a low conversion rate of fructan.

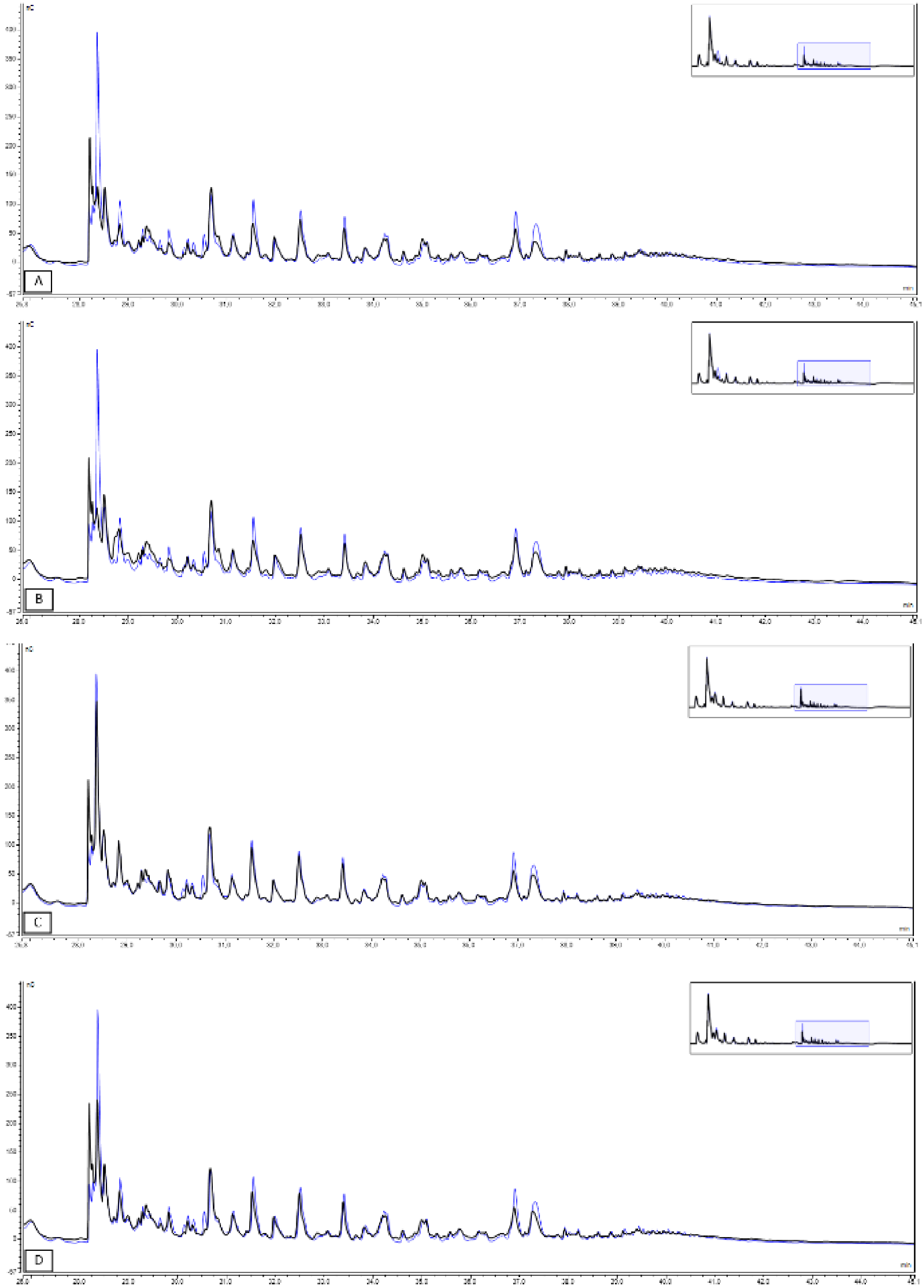
A
B
C
D

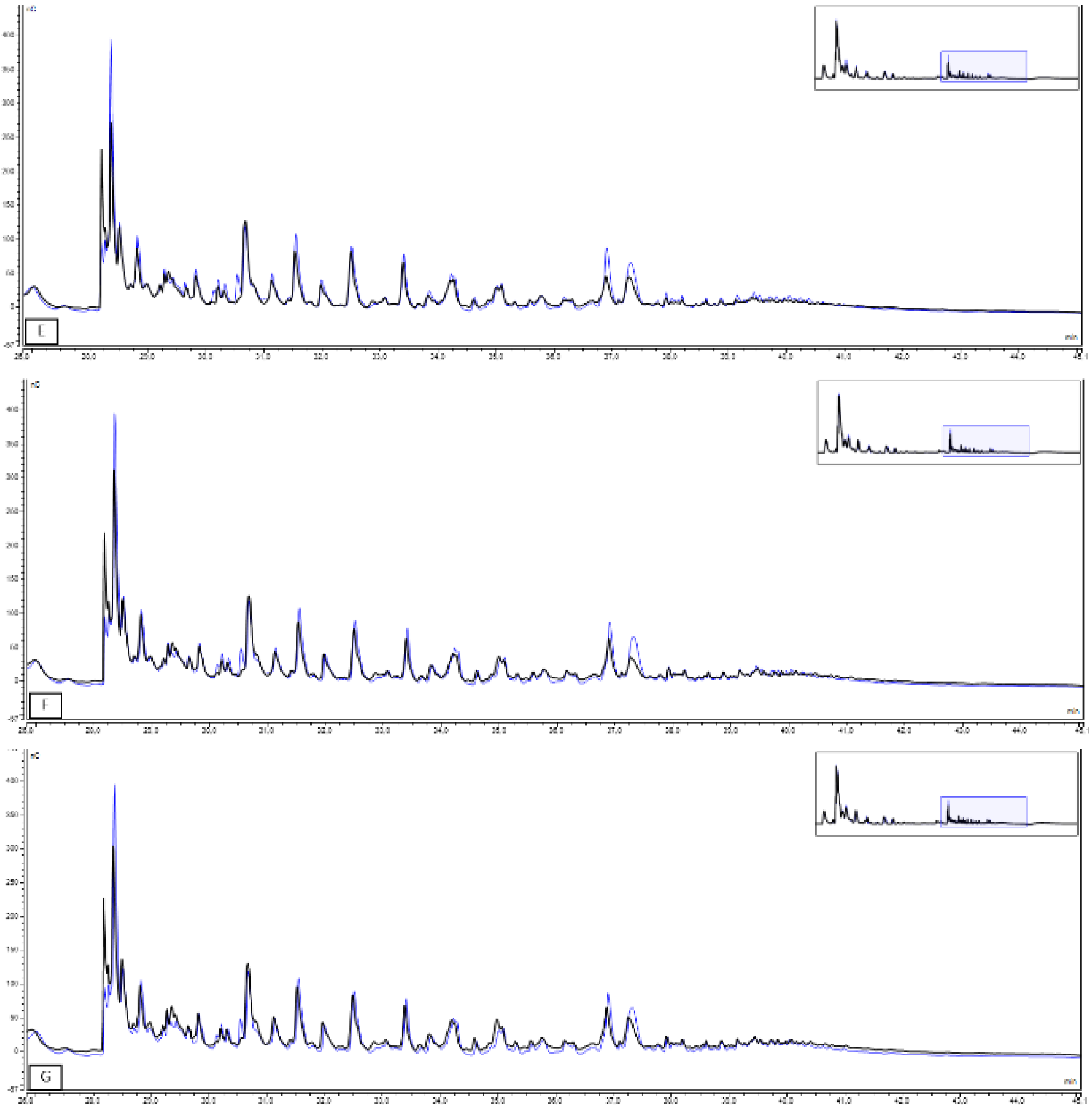

Figure S4: HPAEC-PAD fructan fingerprint analysis of (**A**) *Torulaspora delbrueckii* MUCL 51211, (**B**) *Saccharomyces cerevisiae*–baker's yeast, (**C**) *Kluyveromyces marxianus* MUCL 30016, (**D**) *Wickerhamomyces anomalus* DSM 6766, (**E**) *Torulaspora pretoriensis* MUCL 27827, (**F**) *Candida lambica*–sourdough isolate, and (**G**) *Hanseniaspora uvarum*–sourdough isolate showing the initial fructan presence (blue line) and after 8 h 15 min of fermentation (black line) at 25 °C, with moderate degradation of fructans.

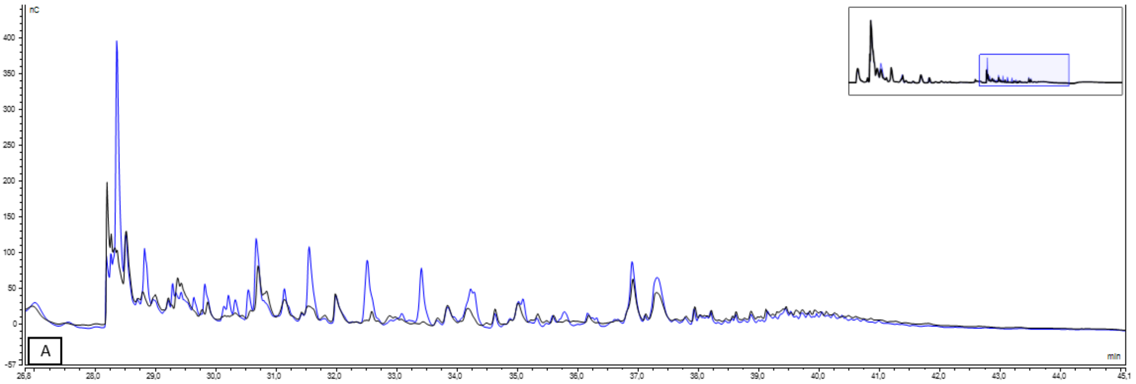

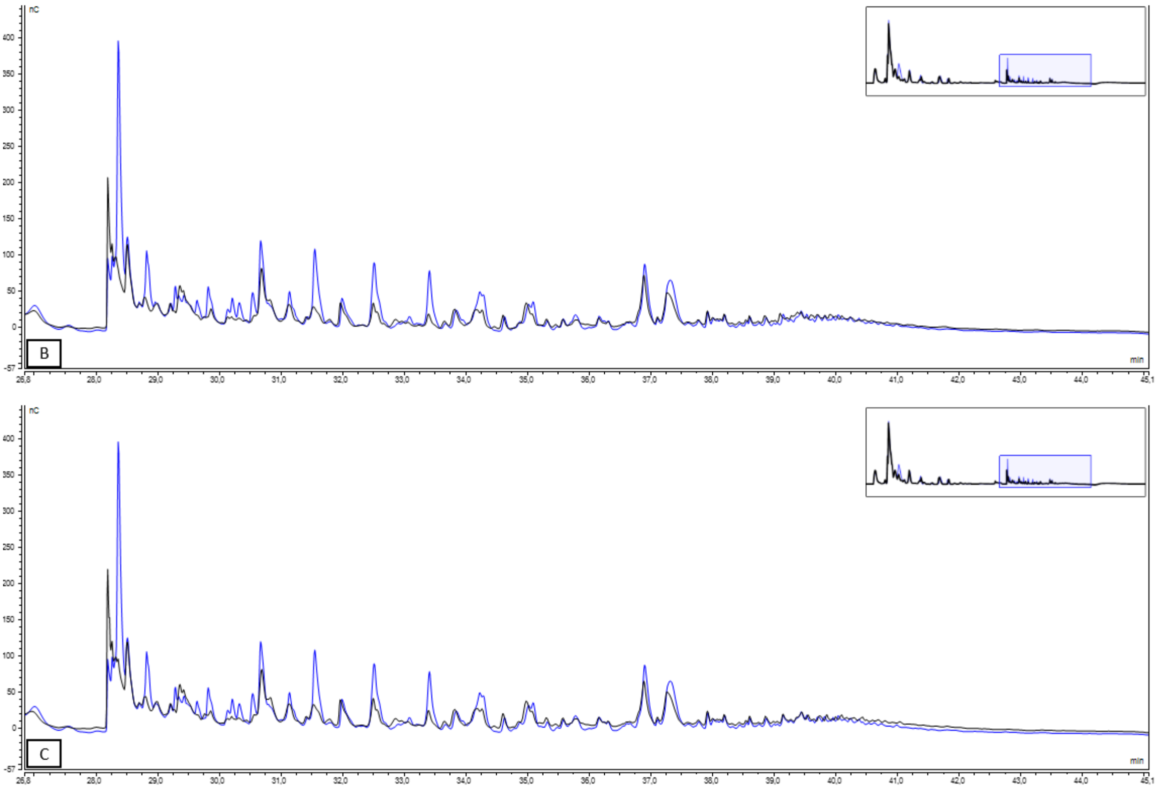

Figure S5: HPAEC-PAD analysis of (**A**) *Kazachstania exigua* MUCL 52365, (**B**) *Saccharomyces cerevisiae*–sourdough isolate, and (**C**) *Torulaspora delbrueckii*–sourdough isolate, showing the initial fructan presence (blue line) and after 8 h 15 min of fermentation (black line) at 25 °C, with the highest degradation of fructans.
